# Motion analysis of the JHU-ISI Gesture and Skill Assessment Working Set using Robotics Video and Motion Assessment Software

**DOI:** 10.1007/s11548-020-02259-z

**Published:** 2020-10-06

**Authors:** Alan Kawarai Lefor, Kanako Harada, Aristotelis Dosis, Mamoru Mitsuishi

**Affiliations:** 1 Department of Bioengineering, School of Engineering, The University of Tokyo, Tokyo, Japan; 2 Department of Mechanical Engineering, School of Engineering, The University of Tokyo, Tokyo, Japan; 3 Imperial College London, London, UK

**Keywords:** Motion analysis, JIGSAWS, ROVIMAS

## Abstract

**Purpose:**

The JIGSAWS dataset is a fixed dataset of robot-assisted surgery kinematic data used to develop predictive models of skill. The purpose of this study is to analyze the relationships of self-defined skill level with global rating scale scores and kinematic data (time, path length and movements) from three exercises (suturing, knot-tying and needle passing) (right and left hands) in the JIGSAWS dataset.

**Methods:**

Global rating scale scores are reported in the JIGSAWS dataset and kinematic data were calculated using ROVIMAS software. Self-defined skill levels are in the dataset (novice, intermediate, expert). Correlation coefficients (global rating scale-skill level and global rating scale-kinematic parameters) were calculated. Kinematic parameters were compared among skill levels.

**Results:**

Global rating scale scores correlated with skill in the knot-tying exercise (*r *= 0.55, *p *= 0.0005). In the suturing exercise, time, path length (left) and movements (left) were significantly different (*p *< 0.05) for novices and experts. For knot-tying, time, path length (right and left) and movements (right) differed significantly for novices and experts. For needle passing, no kinematic parameter was significantly different comparing novices and experts. The only kinematic parameter that correlated with global rating scale scores is time in the knot-tying exercise.

**Conclusion:**

Global rating scale scores weakly correlate with skill level and kinematic parameters. The ability of kinematic parameters to differentiate among self-defined skill levels is inconsistent. Additional data are needed to enhance the dataset and facilitate subset analyses and future model development.

## Introduction

The paradigm for surgical education since the time of Halstead was “see one, do one, teach one” but this has undergone radical change in the last 30 years with the advent of laparoscopic surgery (1987), the Institute of Medicine “To err is human” report (1999) [1] and introduction of the common duty-hour restrictions by the Accreditation Council for Graduate Medical Education (2003). These three watershed events mandated a new surgical education paradigm. The new approach to surgical education is based on objective assessment and obtaining competence, also known as proficiency, instead of subjective assessment that characterizes the Halsteadian paradigm.

Simulation is a cornerstone of surgical and procedural education. Along with changes in teaching, there have been changes in assessment. Simulation allows proficiency-based training, deliberate and distributed practice, which are the three pillars of a surgical curriculum [2]. There have been many attempts to develop objective methods of assessing surgical skill [3, 4]. A variety of global rating scales (GRS) were developed including the OSATS score [5], the GEARS score [6] and GOALS [7] to quantitatively assess skills which depend on assessment by trained observers. Checklists have also been used to assess surgical skills and have been used alone or in combination with GRS [8]. There have been no attempts to quantify the performance of open surgery, other than using a GRS. Skill in open surgery does not necessarily correlate with skill in minimally invasive surgery [3].

Hand motion studies that quantitatively assess the performance of laparoscopic surgery are valid for assessing surgical skill [9–14]. Hand motion studies of simulated procedures are easy to conduct but many not reflect actual surgical skill while studies during laparoscopic surgery are complicated by concerns for the sterile field and the need for sensors to be placed on the hands of operating surgeons [9, 11–13].

Robotic minimally invasive surgery (RMIS) allows the collection of detailed motion data during surgery without concern for the sterile field enabling the collection of more data than from hand motion studies. Metrics of surgical performance in RMIS including time, movements and path length (PL) have been validated and can differentiate novice from expert surgeons [15–18]. RMIS is performed almost exclusively with the da Vinci system (Intuitive Surgical, Sunnyvale, CA, USA). Obtaining motion data from the da Vinci requires approval of the Intuitive Corporation and has been authorized for only a few institutions. Data are delivered according to the format specified in the application programming interface (API) [19]. One of the earliest approaches used to analyze these data is the Robotics Video and Motion Assessment Software (ROVIMAS), developed for this purpose (by one of the authors of this study, AD) [11, 17]. ROVIMAS analyzes data from the da Vinci surgical system and reports time, PL and number of movements and other parameters and has also been used to quantify improved dexterity in RMIS compared with laparoscopic surgery using parameters other than time [20]. Alternatives have been developed to obtain hand motion data during RMIS without the API data [21].

The JHU-ISI Gesture and Skill Assessment Working Set (JIGSAWS) dataset was generated at Johns Hopkins University and is a standardized dataset from simulated RMIS with three exercises (suturing, knot-tying and needle passing) performed by eight participants with varied prior experience [22]. JIGSAWS is the largest publicly available dataset for gesture analysis, and previous work has focused on skill evaluation, gesture classification, gesture segmentation and surgical task recognition [23]. The dataset is fixed and cannot be modified. Since data from one’s own da Vinci system are unavailable to most investigators, the data in JIGSAWS are used to evaluate new models to predict surgical skill. Studies using the JIGSAWS data include an assessment of skill based on video data applied to a convolutional neural network [24], studies of holistic features of the data [25] and gesture analysis [26]. Investigators have used the JIGSAWS dataset to develop predictive models with a deep learning framework, as well as a neural network and a deep neural network which were then used to evaluate study participants [27–29].

The purpose of this study is to examine the relationships of self-defined (SD) skill levels, GRS scores and kinematic parameters in the JIGSAWS dataset. We hypothesized that global rating scale (GRS) scores and/or kinematic parameters correlate with skill level (SD by hours of robotic surgery experience) and can differentiate among the SD skill levels in the JIGSAWS dataset. The correlation of GRS scores with skill levels will be evaluated. For each of the three exercises (suturing, knot-tying and needle passing), kinematic parameters (time, path length and movements) will be calculated from the JIGSAWS dataset using ROVIMAS software. The ability of kinematic parameters to differentiate among skill levels and correlation of kinematic parameters with GRS scores will be evaluated.

## Methods

### JIGSAWS dataset

Three robotic-assisted surgery simulation exercises (suturing, knot-tying and needle passing) were performed on a da Vinci surgical system at Johns Hopkins University [22]. Motion data collected from the da Vinci API were collected and made available online [30]. This study is an analysis of the published dataset.

The dataset includes kinematic data, video data, gestures and a GRS score. Data were collected from participants performing five trials of three exercises (suturing, knot-tying and needle passing) using the da Vinci surgical system. Kinematic data were collected directly from the da Vinci API. The GRS score is a modified OSATS scale assigned during each trial by a trained observer. Global rating scale data are provided as part of the JIGSAWS dataset and require no analysis. The GRS score has six scales including respect for tissue, suture/needle handling, time and motion, flow of operation, overall performance and quality of final product, measured from 1 to 5. [22].

Data were collected from eight participants (referred to in the dataset as B, C, D, E, F, G, H and I), who performed the three exercises. Each performance by a participant is referred to as a trial, for a maximum of 40 trials for each of the three exercises [22]. The developers of the dataset described corruption of data for some trials. Data for these trials are not available. The actual number of trials analyzed for each exercise is shown in Table 1 [22]. SD skill levels were based on participant self-classifications based on hours of experience as novice (< 10 h), intermediate (10–100 h) or expert (> 100 h) operators. There were four novices (B, G, H and I), two intermediates (C and F) and two experts (D and E) based on SD skill levels.

**Table 1:** JIGSAWS dataset global rating scale scores according to skill level and task. Skill level is self-declared by the participant according to criteria in [12]

	Suturing	Knot-tying	Needle passing
Number of trials	39	36	28
Novice	17.5 ± 4.40	10.7 ± 4.19	16.0 ± 5.14
Intermediate	25.1 ± 4.09	17.1 ± 4.28	14.0 ± 6.05
Expert	16.3 ± 3.65	17.7 ± 3.02	12.4 ± 2.35
Correlation of self-declared skill levels with global rating scale scores^a^	*r *= 0.104 *p *= 0.53	*r *= 0.55 *p *= 0.0005	*r *= − 0.293 *p *= 0.13

Global rating scale scores are reported as mean ± standard deviation

^a^Spearman’s correlation

### ROVIMAS

ROVIMAS was developed to analyze data from the da Vinci surgical system and has also been used to evaluate hand motion data from magnetic sensors on the surgeons’ hands in the operating room [12, 17]. ROVIMAS calculates the time for a procedure, the number of movements and PL. Some mathematical notation is needed to define these three parameters which form the basis of motion analysis of RMIS data.

- Time is measured by the clock.
- A single movement is defined as a change in velocity which reaches its maximum as the movement occurs and then returns nearly to zero as the movement is completed [11, 31]. ROVIMAS calculates the distance *d*_*AB*_, between points *A* and *B* in the time interval dt using:

$$ d_{AB} = \sqrt {(x_{B} - x_{A} )^{2} + (y_{B} - y_{A} )^{2} + (z_{B} - z_{A} )^{2} } $$

with (*x*_*A*_, *y*_*A*_, *z*_*A*_) as the coordinates of the first point and (*x*_*B*_, *y*_*B*_, *z*_*B*_) for the second point [11]. The movement pattern is shown by plotting the distance values versus time, and the slope of the resulting line for a movement gives the velocity. This is observed for both sharp and smooth movements. A Gaussian filter is used to smooth the data to differentiate between sudden and controlled movements [11, 17]. The total number of movements is obtained by adding the local high peaks in the smoothed signal [11].

- The total PL of the master controller is calculated by summing all the partial distances [9], where *N* is the number of partial distances and d_i_ is the distance between two neighboring points:
  $$ {\text{PL}} = \sum\limits_{i = 1}^{N} {d_{i} } $$

### Kinematic data

Data in JIGSAWS were recorded at 30 Hz, with 19 data points for each of the four controllers: Right Master, Left Master, Right Slave and Left Slave, resulting in 76 values at each time point as a subset of the 192 values provided by the da Vinci API. ROVIMAS was designed to accept data from version 4.1 of the API [19]. Therefore, the data were converted from the format in the JIGSAWS dataset to the format accepted by ROVIMAS. The conversion was performed by custom software written in Visual C# (Microsoft Corp, Redmond WA USA). Since data were recorded at a constant 30 Hz, the time for each trial was calculated by the number of data points divided by 30, yielding the time for each trial in seconds.

### Statistical analysis

The global rating scale scores and data for time, movements and PL were collected and grouped according to SD skill levels by each participant for all trials of the exercises. Data were compared using the Mann–Whitney *U* test using Excel (Microsoft Corp, Redmond WA USA) and XLSTAT (Addinsoft, Long Island City NY USA). A *p* value of < 0.05 was considered significant. The correlation of continuous variables of time, movements and PL with GRS scores was evaluated using Pearson’s correlation. The correlation of the categorical variable of SD skill level (novice, intermediate, expert) with GRS was evaluated with Spearman’s correlation for each of the three exercises [32]. Correlation is classified as strong (> 0.7), moderate (> 0.5) or weak (> 0.3) [33].

## Results

### Global rating scale score and skill classification

The mean GRS scores comparing the three groups of participants defined by SD skill level are shown in Table 1. The correlation coefficients between the SD skill level (novice, intermediate and expert) and the GRS are shown in Table 1. Of the three exercises, only knot-tying had a significant correlation (*r *= 0.55, *p *= 0.005) between SD skill level and GRS scores.

### Kinematic data

Motion analysis of each of the three exercises is shown in Tables 2 and 3. Correlation of the three kinematic parameters with the self-described skill level is shown in Table 3. Table 3 shows the values for differences in the three kinematic parameters according to skill levels for each exercise based on SD skill level classification. PL and movements are shown for both left and right hands in Tables 2 and 3, including comparisons of all skill levels.

**Table 2:** JIGSAWS dataset motion analysis using ROVIMAS. Skill level is self-declared by the participant according to criteria in [12]

	Time (s)	Right path length (m)	Right movements	Left path length (m)	Left movements
*Suturing*					
Novice 19 trials	137 ± 49.6	0.290 ± 0.230	34.5 ± 25.2	3.02 ± 0.76	40.3 ± 13.5
Intermediate 10 trials	88.5 ± 14.4	0.440 ± 0.130	49.1 ± 14.6	3.10 ± 0.35	63.3 ± 7.45
Expert 10 trials	101 ± 20.0	0.500 ± 0.530	47.8 ± 44.7	1.72 ± 0.25	14.7 ± 4.62
*Knot-tying*					
Novice 16 trials	71.5 ± 18.9	0.16 ± 011	21,8 ± 13.2	1.58 ± 0.66	17.0 ± 9.41
Intermediate 10 trials	47.4 ± 18.8	0.45 ± 0.21	43.5 ± 17.8	2.11 ± 0.81	20.6 ± 7.95
Expert 10 trials	44.0 ± 5.06	0.31 ± 0.12	37.1 ± 13.1	1.02 ± 0.18	13.5 ± 4.14
*Needle passing*					
Novice 11 trials	118.18 ± 16.6	0.26 ± 0.22	31.4 ± 23.6	1.80 ± 0.51	13.3 ± 7.82
Intermediate 8 trials	93.38 ± 30.4	0.50 ± 0.21	50.0 ± 17.4	2.06 ± 0.50	32.5 ± 7.41
Expert 9 trials	108.7 ± 22.2	0.28 ± 0.11	32.1 ± 12.7	1.72 ± 0.32	13.9 ± 4.23

Values shown are the mean ± standard deviation

**Table 3:** Probability values* comparing time, path length, movements and global rating scale (GRS) scores for suturing, knot-tying and needle passing by novice (*N *= 4), intermediate (*N *= 2) and expert (*N *= 2) participants

Exercise	Parameter	Novice/intermediate*	Intermediate/expert*	Novice/expert*	Correlation with GRS**
Suturing	Time	*p *< .05	0.060	*p *< .05	− 0.34
	Global rating scale	*p *< .05	*p *< .05	0.737	–
	Left path length	0.512	*p *< .05	*p *< .05	0.11
	Left movements	*p *< .05	*p *< .05	*p *< .05	0.45
	Right path length	0.120	0.730	0.242	− 0.14
	Right movements	0.215	0.423	0.591	− 0.085
Knot-tying	Time	*p *< .05	0.956	*p *< .05	− 0.69
	Global rating scale	*p *< .05	0.985	*p *< .05	–
	Left path length	0.097	*p *< .05	*p *< .05	− 0.39
	Left movements	0.344	*p *< .05	0.465	− 0.14
	Right path length	*p *< .05	*p *< .05	*p *< .05	0.17
	Right movements	*p *< .05	0.26	*p *< .05	0.34
Needle passing	Time	0.104	0.409	0.157	− 0.30
	Global rating scale	0.503	0.901	0.083	–
	Left path length	0.492	0.167	0.656	0.19
	Left movements	*p *< .05	*p *< .05	0.641	− 0.015
	Right path length	*p *< .05	*p *< .05	0.417	− 0.15
	Right movements	*p *< .05	*p *< .05	0.86	− 0.17

Skill level is self-declared by the participant according to criteria in [12]. Data for both hands are shown

*Probability values (*p* values), Mann–Whitney *U* test

**Pearson correlation coefficient

### Suturing exercise

There is a significant difference between novices and experts for PL (*p *< 0.0001) movements (*p *< 0.0001) and time (*p *= 0.012) for the left hand but not the right hand. Movements are the most consistent among the three parameters tested being significantly different among all three skill levels for the left hand, but not for the right hand.

Time and movements weakly correlate with GRS scores (*r *= − 0.34 and 0.45, respectively). The correlation of movements with GRS scores is positive for the left hand and negative for the right hand. The GRS scores are significantly different between the intermediate level and both novice and expert levels.

**Fig. 1:**
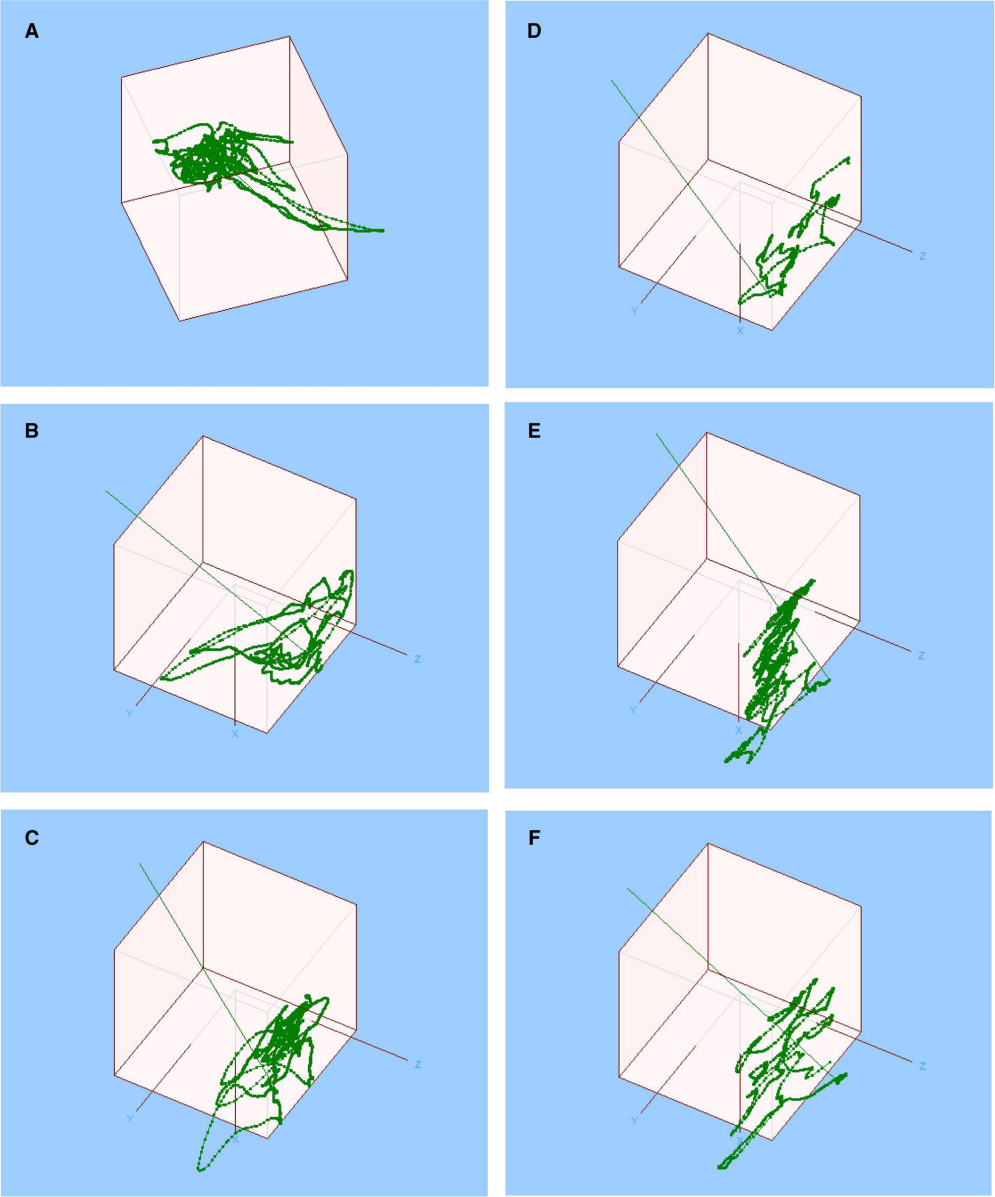
Three-dimensional Cartesian trajectory analysis (left hand is shown in all graphs) provided by ROVIMAS shows that participants classified as experts have fewer and more focused trajectories than novices, similar to the patterns reported by others [14, 21, 34]. The origin of each graph is defined by the initial position of the instruments of the da Vinci surgical system at startup and the positions of the instrument tip shown. **a**, **b** Trajectory analysis of the suturing exercise completed by participants B and E, self-described as a novice and expert, respectively. **c**, **d**. Trajectory analysis of the knot-tying exercise completed by participants I and D, self-described as a novice and expert, respectively. **e**, **f** Trajectory analysis of the needle passing exercise completed by participants I and D, self-described as a novice and expert, respectively

ROVIMAS provides trajectory analysis and representative analyses are shown for a novice participant (Fig. 1a) and an expert (Fig. 1b) in the suturing exercise.

### Knot-tying exercise

Table 3 shows that there is a significant difference for time (*p *< 0.0001) and PL (*p *= 0.045) comparing novice and expert SD skill levels. Similar to the suturing exercise, there is no pattern maintained for differences in significance comparing the left and right hands. Movements are significantly different between novices and experts for the right hand but not the left hand.

There is a moderate correlation between time and GRS score (*r *= − 0.69). There is a significant difference for GRS scores comparing expert/novice operators and novice/intermediate operators. Left hand kinematic parameters have a negative correlation with GRS, while right hand parameters have a positive correlation, showing again that there is no consistent pattern of differences between left and right hands.

**Fig. 2:**
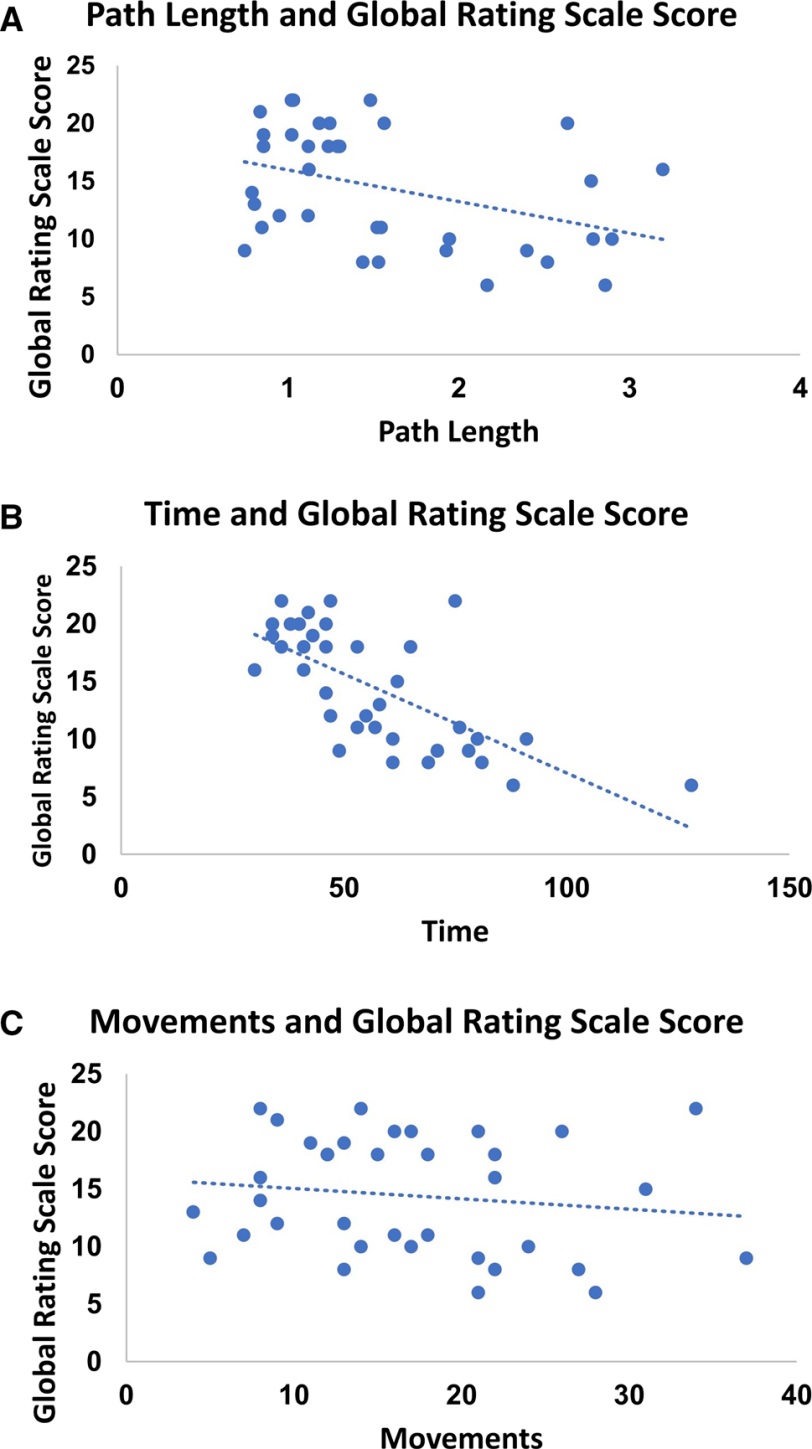
Representative scatter plots of path length (m) (**a**, *r *= − 0.36), time (s) (**b**, *r *= − 0.65) and movements (**c**, *r *= − 0.33) versus global rating scores for the knot-tying exercise. Linear trend lines are shown in each figure

Representative trajectory analyses are shown for a novice participant (Fig. 1c) and an expert (Fig. 1d) in the knot-tying exercise. Representative scatter plots of PL (Fig. 2a), time (Fig. 2b) and movements (Fig. 2c) versus global rating scores are shown for the knot-tying exercise which show moderate correlation of GRS with time in this exercise.

### Needle passing exercise

Of the three kinematic parameters, there are significant differences for movements comparing intermediate/novice and intermediate/expert operators for the left hand and right hand. There are no significant differences comparing skill levels for PL or time for the left hand but there are differences for novice/intermediate and intermediate/expert for the right hand.

There are no significant differences comparing GRS scores among the skill levels, and GRS scores correlate weakly with the kinematic parameters for both left and right hands with no specific pattern in the sign of the correlation.

Representative trajectory analyses are shown for a novice participant (Fig. 1e) and an expert (Fig. 1f) for the needle passing exercise.

## Discussion

Time, PL and number of movements have been validated as kinematic parameters for the assessment of laparoscopic surgical skills [14]. These three kinematic parameters were evaluated for the eight participants in the three exercises (suturing, knot-tying and needle passing) in the JIGSAWS dataset using ROVIMAS software as well as the GRS for each trial of the three exercises.

Previous studies have examined the correlation between hand motion and surgical skill [9, 10, 12, 30, 34]. Hand motion has also been used in the training of anesthesiologists [35]. Motion tracking devices have been attached to surgeons’ hands during actual surgery and the data analyzed by ROVIMAS [12]. This study found differences in surgeons with different skill levels for time, PL and number of movements. Hand motion studies have also been done in a simulation environment [9, 10]. Similar differences in trajectory analysis were also reported by others [16, 19, 23, 36]. Trajectory analysis in these studies showed results similar to those in the present study for the JIGSAWS data (Fig. 1), that experts have a more focused trajectory.

A partial motion analysis of the JIGSAWS dataset has been reported [16]. These investigators analyzed the suturing exercise and the knot-tying exercise but did not discuss the needle passing exercise and used a different definition of novice and expert operators based on GRS scores. Data in that study show that motion analysis of the left hand (nondominant for all JIGSAWS participants) is more important than data from the right hand, and that dexterity can be assessed based on nondominant hand performance. All participants in the JIGSAWS dataset were right-hand dominant. The correlation of kinematic parameters with GRS should be negative, but in the suturing exercise, left hand parameters have a positive correlation with GRS, while right hand parameters have a negative correlation (Table 3). There is no consistent correlation between kinematic parameters and GRS for either hand. Similarly, differences in significance of kinematic parameters between skill levels are not consistent regarding the left or right hands. These results suggest that data for both hands should be evaluated.

ROVIMAS analysis in this study using SD skill levels shows that the PL for novices was longer than for experts (Table 3). In a previous partial analysis of the JIGSAWS dataset, the PL for the left hand was slightly longer for experts than novices in the suturing exercise [16]. In the present study, the PL is slightly shorter for experts. This may be due to differences in the software used for analysis. A deep surgical skill classification model was developed which used SD skill classification [27]. Other studies developed models using both classifications and showed nearly equal results [28]. Other predictive models are based on the SD classification [23, 25, 29]. These studies used kinematic data without motion analysis.

The correlations of the three kinematic parameters with GRS scores are generally weak in all three exercises in this analysis (Table 3). The trend lines show a weak correlation (Fig. 2), which is overall best for the time analysis in all three exercises. A similar observation was made using data from a clinical study [15]. Fard and colleagues stated that time and PL are insufficient to explain all aspects of surgical assessment [16]. In the suturing exercise, they computed a correlation coefficient for time of 0.43 and PL of 0.27. Others have reported that all objective kinematic parameters evaluated including time and PL can distinguish between novice and expert performance [18]. The differences in PL calculation between this study and previously published results are acknowledged [16]. The reason for this difference is unclear and difficult to explain, especially since the software from the other study is not available. However, despite this difference, we believe that results within this study, all of which were calculated with ROVIMAS, are a valid basis of comparison.

The results of the knot-tying exercise are interesting because there is a small difference in GRS scores between intermediate and expert participants (Table 1, 17.1 and 17.7, respectively), which was used to explain poor skill classification performance for this exercise [23]. Despite this, there is a moderate correlation between GRS score and time in this exercise in the present analysis. The intermediate skill level may be difficult to interpret. First, we expect the greatest differences to be between novice and expert participants so these data may show a greater difference. Using novice and expert classifications alone reduces the problem to a binary classification [16].

There are acknowledged limitations to this study. The data provided in the JIGSAWS dataset and are used “as is” so that any limitations in the data or methodology are inherent in this study. The JIGSAWS dataset is limited in size which limits the extent of this study as well as limiting the ability to conduct appropriately powered subset analyses. ROVIMAS cannot directly read the data in the JIGSAWS dataset, and there is always a chance of data corruption in the conversion process. Due to software limitations, it is not possible to modify the source code of ROVIMAS and add desired features.

It has been said that “It is somewhat surprising that there are no tools in widespread use that are feasible, valid, and reliable for assessment of technical surgical skill” [12]. The “holy grail” of surgical assessment is a single tool which can accurately evaluate surgical skill. It remains to be shown that such assessments are clinically relevant [23]. It is also unknown whether simulation education results in improved clinical performance in robot-assisted surgery, in contrast to laparoscopic surgery [37]. Objective assessment of clinical surgical skill remains an elusive goal, in part because it has not been possible to demonstrate a clear linkage between such assessments and clinical performance partly because clinical outcomes depend on a wide range of factors attributable to both surgeon and patient.

The relationship between kinematic parameters and surgical skill appears to be nonlinear and will need further refinement of analytical tools to conduct nonlinear analyses, such as a deep learning approach which has been performed by some investigators [27–29]. There is no shortage of assessment tools, but assessment of surgical skill remains a complex and difficult task to perform in a meaningful way [3, 4]. It is reasonable to suggest that assessing surgical skill in RMIS requires multiple simultaneous assessments including global rating scales (such as GEARS, OSATS), gesture analysis and motion analysis.

## Conclusions

This study shows weak correlation of GRS scores with SD skill level for suturing and needle passing, and moderate correlation for knot-tying. Kinematic parameters do not correlate strongly with GRS scores as one measure of skill, and while some parameters can differentiate among different SD skill levels, no one parameter consistently makes this differentiation. The JIGSAWS dataset is of great importance in studies of robotic-assisted surgery kinematic data because it is publicly available and obtaining surgical robot motion data may not otherwise be possible. This study provides further insight into this dataset that is being used to develop models to predict surgical skill. This dataset may be enhanced by including more participants and more trials to allow appropriately powered subset analyses. These results should be considered in the development of future assessment tools.

## Data Availability

All data is available online [30].
